# Intense vortical-field generation using coherent superposition of multiple vortex beams

**DOI:** 10.1038/s41598-023-28216-9

**Published:** 2023-01-20

**Authors:** Xinju Guo, Xiaomei Zhang, Dirui Xu, Weixin Chen, Yi Guo, Ke Lan, Baifei Shen

**Affiliations:** 1 Department of Physics, Shanghai Normal University, Shanghai, 200234 China; 2 Institute of Applied Physics and Computational Mathematics, Beijing, 100094 China; 3 HEDPS, Center for Applied Physics and Technology, and College of Engineering, Peking University, Beijing, 100871 China

**Keywords:** Optics and photonics, Physics

## Abstract

Coherent beam combining technology applied to multiple vortex beams is a promising method to generate high-power vortex beams. We utilize the coherent combination of multiple Laguerre-Gaussian beams at the waist plane and propose theoretically a practical generation system for a high-power beam carrying orbital angular momentum by considering oblique incidence. The results demonstrate that the orbital angular momentum distribution of the combined field is similar to that of a single Laguerre-Gaussian beam within the Rayleigh length. Moreover, the combined field has relativistic intensity local spots that exhibit stable spatial propagation. The proposed system may potentially be applied to intense vortical fields, large scale nuclear fusion device, such as suppressing stimulated Raman scattering and filamentation when a laser beam propagates in plasma.

## Introduction

The vortex beam is a special beam that carries orbital angular momentum (OAM). It features helical phase-front twisting as it propagates, leading to a null in the field amplitude and a singularity in the field phase^1,2^. They have found numerous applications in a variety of classical and quantum systems^3–5^. In recent years, many studies have attempted to generate vortex beams with higher intensity and better performance^6^. To date, two experimental methods have been mainly used to generate vortex beams: direct generation in the laser cavity^7–9^ and indirect conversion based on a plane wave using various phase adjustors^10–16^. However, it is difficult to further improve the output power of vortex beams to relativistic intensity due to the limited power-handling capacity of available phase adjustors^17,18^, the challenges of nonlinear optical effects^19^, and the thermal effect that occurs during the power scaling of single-channel laser beams. Notably, high-intensity vortex beams are required in an increasing number of fields, such as nonlinear frequency-conversion processes^20^, satellite-to-ground communication^21^, laser ablation^22^, material processing^23^ and many other high-energy physics applications^24^. Therefore, it is necessary to develop an alternative approach to overcome the limitations of single lasers and access higher-power vortex beams. Few approaches can generate high-intensity vortex beams, e.g., based on advanced laser-plasma interaction schemes, there has been proposed theoretically and demonstrated experimentally, including the interaction of ultra-intense laser vortices with plasma mirrors^25,26^, transient plasma holograms^27^, or using off-axis spiral phase mirrors to generate high-intensity optical vortices^28^.

At present, coherent beam combining (CBC)^29–33^ is widely used to produce higher-intensity lasers that can surpass the power limitation of a single laser beam. Previously, the CBC of a fiber laser array was used to increase the average output power to kilowatts while ensuring good performance^34–37^. Many previous studies have attempted to generate high-intensity OAM beams using the coherent combination of a laser array^38–45^. One method to generate a vortex beam is to use the CBC of sub-Gaussian beams. In the experiment, the high-intensity vortex beam was realized by the addition of a helical sub-beam phase and phase locking^45^. Moreover, in theoretical research, the vortex laser power was considerably improved by adjusting the arrangement of the Gaussian beams at the source field, the number of sub-beams, phase-control approaches, and the introduction of feedback devices^43^. The propagation characteristics of the vortex beam, the OAM density distribution, and the intensity distribution of the combined field in the superposition process have also been investigated^46–49^. The second method to generate vortex beams is to use sub-beams of coaxial coherent superposition as vortex beams, which is still in the theoretical stage of study. The combined field of beams that carry OAM will be more complex owing to the helical wavefront structure; however, this may enable the realization of many new characteristics. For example, by adjusting basic parameters, such as the frequency, topological charge, and the radial index of the incident sub-beam, spatiotemporal beams containing two independent and controllable OAMs can be realized^40^. Using a combination of multiple-mode orthogonal vortex beams with complex coefficients, a beam structure containing a high-power-density local spot can be readily achieved in the azimuthal direction^42^. However, the laser intensity is limited owing to the use of transmissive optical elements.

In this study, we propose theoretically a practical method to generate a high-power beam carrying OAM by considering the use of oblique incidence for the sub-vortex beams. Therefore, the sub-vortex beams are non-coaxial. Our results show that the average OAM per photon in the combined vortex field is equal to that of the incident light. Moreover, the radial OAM distribution of the combined field is similar to that of the incident Laguerre-Gaussian (LG) beam. The interference of the sub-beams causes several high-intensity local spots in the transverse plane, which can spatially propagate stably within the Rayleigh distance. The peak intensity of the combined field is close to ${N}^{2}{I}_{0}$ (*N* is the number of sub-beams, and ${I}_{0}$ is the peak intensity of the single sub-beam). Here we should note that our work presents the first implementation of a constructed vortex beam based on non-coaxial coherent beam combining in an intense field and it is also the first theoretical study, which is based on the similar layout and parameters of existing large-scale fusion devices. This work provides helpful guidance for generating high-intensity vortex light field in practical experiments, and the potential applications are as follows: first, the vortex laser obtained by oblique-incidence LG coherent beam combination can improve the intensity of vortex beam and produce relativistic vortex laser in practical experiments; Second, the relativistic intensity laser produced is promising to play an important role in the field of large-scale nuclear fusion, such as suppressing stimulated Raman scattering and filamentation when a laser beam propagates in plasma.

## Theoretical methods and models

**Figure 1:**
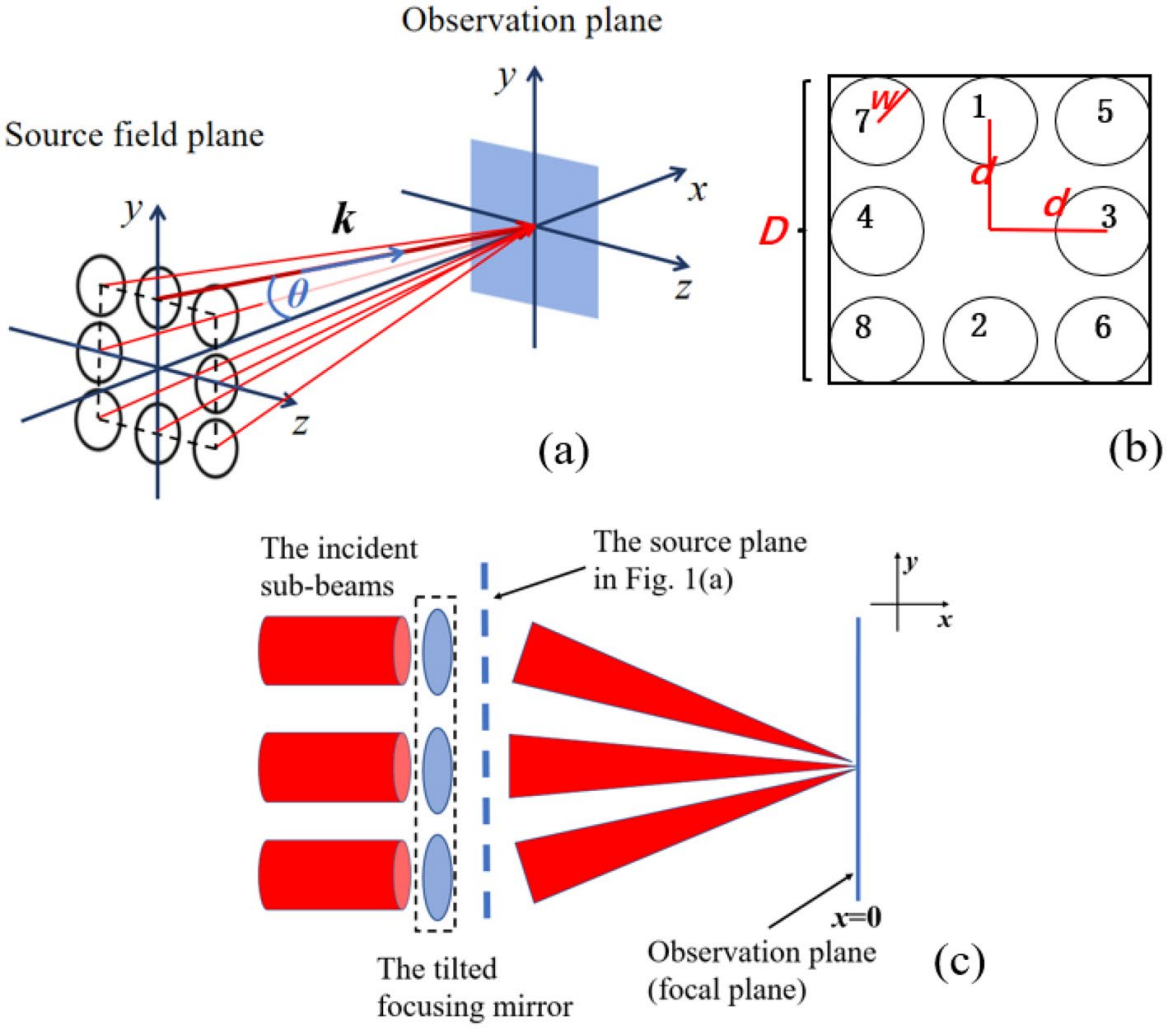
(**a**) Schematic for the generation of intense OAM beams with a coherent laser array, in which the sub beam is incident at an angle *θ* relative to the *x*-axis, and ***k*** is the wave vector of the incident beam. (**b**) Arrangement of incident sub-beams at the source field, where *D* is the diameter of the incident window, *w* is the beam radius of each beam in the incident window, *d* is the distance between adjacent beams, and the number represents the serial number of each beam. (**c**) The simple Experimental Schematic Diagram of Beam Oblique.

We consider an ideal LG beam propagating along the *x*-axis. The spatial distribution of this beam is given by the following expression^1^:

1 $$\begin{aligned} & E\left( {x,y,z} \right) = \left( { - 1} \right)^{p} \frac{{C_{pl} }}{w\left( x \right)} \cdot \left( {\frac{\sqrt 2 r}{{w\left( x \right)}}} \right)^{\left| l \right|} \cdot \exp \left( { - \frac{{r^{2} }}{{w\left( x \right)^{2} }}} \right) \cdot L_{p}^{\left| l \right|} \left( {\frac{{2r^{2} }}{{w\left( x \right)^{2} }}} \right) \\ & \quad exp\left\{ { - i\left[ {\frac{{k \cdot r^{2} }}{{2R_{\mathrm{x}} }} - \left( {2p + \left| l \right| + 1} \right) \cdot \tan^{ - 1} \left( {\frac{x}{{x_{R} }}} \right)} \right]} \right\} \cdot \exp \left[ {ikx + il\varphi + \phi } \right] \\ \end{aligned}$$

with $r=\sqrt{{y}^{2}+{z}^{2}}$, $\varphi = {tan}^{-1}(\frac{z}{y})$, $w={w}_{0}\sqrt{1+\frac{{x}^{2}}{{x}_{R}^{2}}}, {R}_{\mathrm{x}}=\frac{{x}^{2}+{x}_{\mathrm{R}}^{2}}{{x}^{2}}$, where ${C}_{pl}$ is the normalization constant, ${L}_{p}^{\left|l\right|}(x)$ is the associated Laguerre polynomial, and *p* is the number of radial nodes in the intensity distribution, $k=2\pi /\lambda$ is the wave number, ${w}_{0}$ is the beam-waist radius, ${x}_{\mathrm{R}}$ is the beam Rayleigh distance, $l$ is the topological charge, and $\phi$ is the original phase at the waist plane. At the source plane, *N* linearly polarized ${\mathrm{LG}}_{01}$ beams are arranged as sub-beams with the same intensity distribution and waist size in a square domain. The waist center of the sub-beams is at the origin on the *x* = 0 plane. All incident sub beams are in-phase distribution. If all the laser array elements are phase-locked and coherently combined, $\phi$ = 0 can be assumed for the sub-beams at the waist plane. As an example, a combined-beam diagram is shown in Fig. 1a. The sub-beams are incident at an oblique angle *θ*, which is the angle between the wave vector **k** and the *x*-axis.

The incident angle *θ* of each sub-beam differs slightly. Figure 1b shows the transverse distribution diagram of the sub-beams at the source field, where the number represents the serial number of each beam. In the numerical calculation, the oblique-incidence of the beam is realized by rotating the beam propagating along the *x*-axis (defined in Eq. 1) around the *y*-axis and *z*-axis. The rotation angle of each sub-beam around the *y* and *z* axes are written as ${\theta }_{\mathrm{y}}$, ${\theta }_{\mathrm{z}}$, respectively. $\theta =\sqrt{{\theta }_{\mathrm{y}}^{2}+{\theta }_{\mathrm{z}}^{2}}$, and the specific rotation angle is determined by the spatial position of the sub-beam in the actual experiment. For rotating the sub-beam around the *y* and *z* axes, the original coordinate system of the sub-beam needs to be multiplied by a two-dimensional transformation matrix, which can be expressed as

2 $$\begin{array}{*{20}c} {T = \left[ {\begin{array}{*{20}l} {{\text{cos}}\theta_{{\text{z}}} {\text{cos}}\theta_{{\text{y}}} } \hfill & {\quad {\text{sin}}\theta_{{\text{z}}} } \hfill & {\quad - {\text{cos}}\theta_{{\text{z}}} {\text{sin}}\theta_{{\text{y}}} } \hfill \\ {{\text{sin}}\theta_{{\text{z}}} {\text{cos}}\theta_{{\text{y}}} } \hfill & {\quad {\text{cos}}\theta_{{\text{z}}} } \hfill & {\quad {\text{sin}}\theta_{{\text{z}}} {\text{sin}}\theta_{{\text{y}}} } \hfill \\ {{\text{sin}}\theta_{{\text{y}}} } \hfill & {\quad 0} \hfill & {\quad {\text{cos}}\theta_{{\text{y}}} } \hfill \\ \end{array} } \right].} \\ \end{array}$$

The coherent field at the beam-waist plane is $E\left(x,y,z\right)=\sum_{i=1}^{N}[{E}_{i}{\left(x,y,z\right)}_{i}]$, where

$$\left( {\begin{array}{*{20}c} x \\ y \\ z \\ \end{array} } \right)_{i} = T_{i} \cdot\left( {\begin{array}{*{20}c} x \\ y \\ z \\ \end{array} } \right)$$.

The intensity distribution can be represented by

3 $$\begin{array}{*{20}c} {I = \left[ {\sum\nolimits_{i = 1}^{N} {E_{i} \left( {x,y,z} \right)_{i} } } \right]\left[ {\sum\nolimits_{i = 1}^{N} {E_{i} \left( {x,y,z} \right)_{i} } } \right]^{*} .} \\ \end{array}$$

Here the basic experimental assumption can be seen in the following Fig. 1c. The coherent light of different radiation sources is coherent after phase control. The sub-beams mentioned have been focused by a tilted focusing mirror. The focal plane of the sub-beam is in the *x* = 0 plane. Then the sub-beams are oblique incidence into the observation plane (focal plane). The source field shown in Fig. 1a refers to the plane behind the focusing mirror.

The incident angle of the sub-beams should be considered carefully in the theoretical analysis to avoid the overlapping of the sub-beams at the source plane during practical implementation. By calculating the fill factor of the combined field, we determined the relationship between the incident angle and the beam-divergence angle of the sub-beam. In a real experiment, we consider the source plane may be far away from the waist plane, and the beam size at the source plane may be considerably larger so that the intensity is low enough to avoid damaging the mirrors. Here, we assume the source field of the sub beam is much larger than the Rayleigh distance of the sub beam, i.e. *x* >> ${x}_{\mathrm{R}}$, in which the beam radius of the sub-beam is *w*, and the diameter of the incident window is *D*, the sub-beams are arranged with their nearest neighbors separated by a distance *d*, as shown in Fig. 1b. The half-divergence angle ${\theta }_{\mathrm{di}}$ and incident angle ${\uptheta }_{\mathrm{axis}}$ (${\uptheta }_{\mathrm{corner}}$) of the sub-beam at the axis (corner) can be represented by

4 $$\theta_{{{\text{di}}}} = \mathop {\lim }\limits_{x \to \infty } \frac{w}{x} = \mathop {\lim }\limits_{x \to \infty } \frac{{w_{0} \sqrt {1 + \frac{{x^{2} }}{{x_{{\text{R}}}^{2} }}} }}{x} \approx \frac{\lambda }{{{\uppi } \cdot w_{0} }}$$

5 $$\begin{array}{*{20}c} {w = w_{0} \sqrt {1 + \frac{{x^{2} }}{{x_{{\text{R}}}^{2} }}} \approx x \cdot \frac{\lambda }{{\pi \cdot w_{0} }} = x \cdot \theta_{{{\text{di}}}} } \\ \end{array}$$

6 $$\begin{array}{*{20}c} {{\uptheta }_{\mathrm{axis}} \approx \frac{d}{x}, {\uptheta }_{\mathrm{corner}} \approx \frac{\sqrt 2 d}{x}} \\ \end{array}$$

7 $$\begin{array}{*{20}c} {D = 2d + 2w.} \\ \end{array}$$

If the ratio of the divergence angle to the incident angle, ${\theta }_{\mathrm{di}}/{\uptheta }_{\mathrm{axis}}$, is less than 0.5, each sub-beam will be non-overlapping at the source plane.

Additionally, the fill factor at the source field can be calculated as follows:

8 $$\begin{array}{*{20}c} {f = \frac{{8\pi \cdot w^{2} }}{{D^{2} }} = 2\pi \cdot \left( {\frac{{\theta_{{{\text{di}}}} }}{{\theta_{\mathrm{axis}} + \theta_{{{\text{di}}}} }}} \right)^{2} < \frac{2\pi }{9}.} \\ \end{array}$$

## Numerical results and analysis

**Figure 2:**
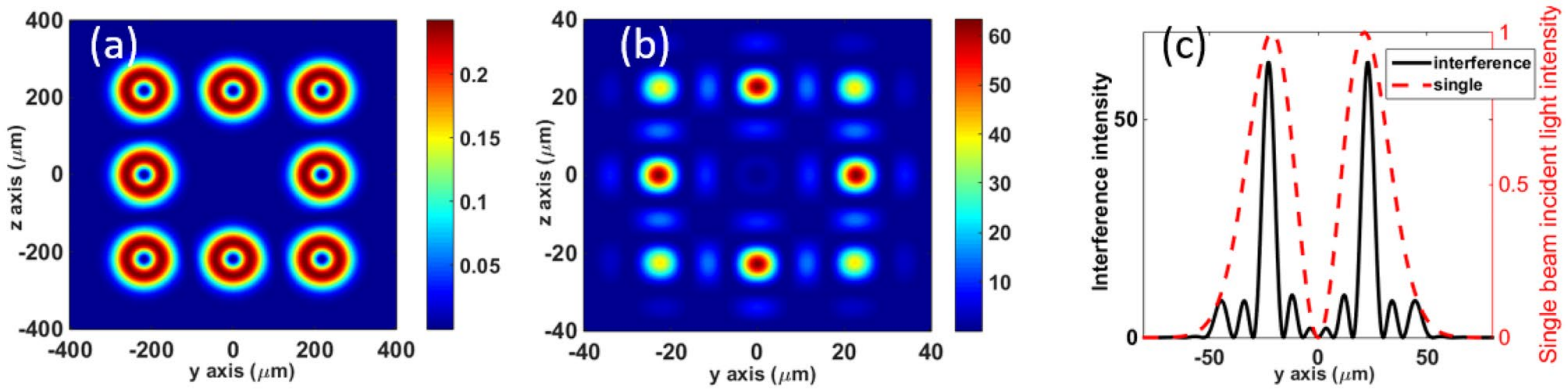
Numerical results. The peak intensity ${I}_{0}$ of the sub-beams is set to 1 at the waist plane *x* = 0. (**a**) Intensity distributions of the sub-beams at the source plane *x* = − 5 mm. See the Supplementary Fig. S1a for the phase distributions of the sub-beams at the source plane. (**b**) Intensity distributions of vortex light combined field at the waist plane *x* = 0. The energy keeps to accumulate towards four local spots during the propagation, which can be observed from the evolution process of the combined field (see the video of Supplementary Material 2). The peak intensity of the combined field is higher than 60${I}_{0}$. See the Supplementary Fig. S1b for the phase distributions of the sub-beams at the waist plane. (**c**) Intensity distribution of the combined field on the *y*-axis at *z* = 0 at the waist plane.

The incident angles of the sub-beams in the numerical calculation, considering the actual experimental requirements, are shown in Table 1. The condition that the ratio of the divergence angle to the incident angle, ${\theta }_{\mathrm{di}}/{\theta }_{\mathrm{axis}}$, is less than 0.5 is satisfied. The source plane of the sub-beams is at *x* = − 5 mm, and the waist plane is at *x* = 0. In a real experiment, the source plane may be far away, and the beam size may be set considerably larger to avoid damage to the mirrors owing to the intense laser beams. The waist radius of the sub-beam is 30 μm, the topological charge *l* = 1, and the normalized peak intensity of each sub-beam at the beam waist plane ${I}_{0}$= 1. Here, all the incident sub-beams are regarded as having infinite duration and constant intensity. The transverse beam intensity distribution of the source plane is shown in Fig. 2a. Figure 2b shows the intensity distribution of the combined field observed at the waist plane. It can be observed that there are four main local spots with high intensity, owing to the interference of the sub-beams. These four local spots appear and gradually strengthen within the Rayleigh distance of the combined field. The energy keeps to accumulate towards these four local spots during the propagation, which can be observed from the evolution process of the combined field (see Visualization 1). The peak intensity of the combined field is approximately 62${I}_{0}$, which is almost the highest intensity of each high-intensity regions. According to interference theory^29^, when eight beams are perfectly coherent, the maximum coherent intensity is $64I_{0}$. Therefore, the maximum intensity of the combined field is close to the perfectly coherent intensity. In addition, the intensity distribution along the *y*-axis at *z* = 0 at the waist plane is shown in Fig. 2c. The red dotted and blue solid lines represent the intensity envelopes of a single-incident LG beam and combined field, respectively. It is clear that the radial position of the peak intensity after coherent superposition is approximately the same as that of a single incident sub-beam. However, compared with the waist radius of the sub-beam ${w}_{0}$ = 30 $\mathrm{\mu m}$, the radii of the high-intensity local spots generated by coherent superposition decrease sharply to 7.7 $\mathrm{\mu m}$. That is, as the intensity increases, the energy becomes more concentrated.

**Table 1:** Incident angles of the sub-beams.

Serial number of sub-beams		1	2	3	4	5	6	7	8
Angle	${\theta }_{\mathrm{y}}$	0°	0°	− 2.5°	2.5°	− 2.5°	2.5°	− 2.5°	2.5°
	${\theta }_{\mathrm{z}}$	− 2.5°	2.5°	0°	0°	− 2.5°	− 2.5°	2.5°	2.5°
	$\theta$	2.5°	2.5°	2.5°	2.5°	2.5°$\cdot \sqrt{2}$	2.5°$\cdot \sqrt{2}$	2.5°$\cdot \sqrt{2}$	2.5°$\cdot \sqrt{2}$

${\theta }_{\mathrm{y}}$ and ${\theta }_{\mathrm{z}}$ are the rotation angles of the sub-beam around the *y* and *z* axes, respectively. We specify that, if the rotation direction of the sub-beam is clockwise, the rotation angle is positive, and vice versa. $\theta$ is the angle between the wave vector ***k*** of a sub-beam and the *x*-axis, $\theta = \sqrt{{\theta }_{\mathrm{y}}^{2}+{\theta }_{\mathrm{z}}^{2}}$. The incident angle of the sub beams at the corner has a multiplier factor of sqrt (2) compared to the beam at the axis due to different layout positions.

**Figure 3:**
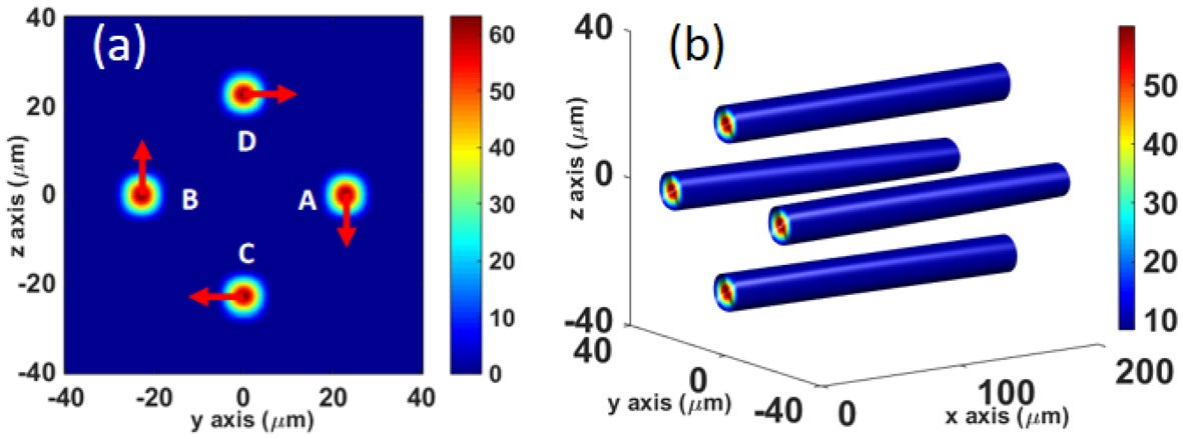
(**a**) Intensity distribution of high-intensity local spots of the combined field at the beam-waist plane, where the field of the other positions is filtered out. (**b**) The isosurface distribution of the high-intensity local spots of the combined field within the Rayleigh distance, where the isosurface value is ${I}_{\mathrm{max}}$·exp(− 2), and ${I}_{\mathrm{max}}$ is the peak intensity of the combined field.

In the following, we focus on the angular momentum of the combined field. The calculation results confirm that the average angular momentum per photon in the total combined field is the same as that of the incident sub-beam, which is still 1 ($\mathrm{\hslash }$ is regarded as 1 in this study) although the intensity distribution is not a perfect donut for ideal LG pulse. We focus on the four high-intensity local spots because they are more promising for potential applications. Therefore, we filter them, as shown in Fig. 3a. As shown in Table 2, we calculate the angular momentum of the four high-intensity local spots. The results show that the average OAM per photon in the four high intensity local spots is 1. In particular, the OAM of the high-intensity region accounts for approximately 44.5% of the total combined-field OAM, which is the same as the energy ratio. In addition, the average OAM per photon in the other part of the combined field is also 1, which is as expected; the sum of the OAM of the high- and low-intensity regions is the same as that of the total combined field. The detailed OAM calculation process can find in Section B of the Supplementary Material.

**Table 2:** OAM calculation of different regions.

	OAM of the four high-intensity local spots	OAM of the local spot A	
Calculated coordinate	*x*-axis	*x*-axis	Mass axis
${j}_{\mathrm{x}}$	− 0.99	− 0.99	0
${j}_{\mathrm{y}}$	0	− 0.11	0
${j}_{\mathrm{z}}$	0	− 143.48	0

$\mathrm{\hslash }$ is regarded as 1 in this study.

The OAM of local spot A, noted in Fig. 3a, is calculated to study the OAM distribution of the combined field more carefully. When the calculation axis is selected at *x*-axis, it is observed that the average OAM per photon is ${j}_{\mathrm{x}}$ = − 1, ${j}_{\mathrm{y}}$ = 0.1, ${j}_{\mathrm{z}}$ = − 143, which exists as an extrinsic OAM in the *z*-axis direction. We then check the OAM of the other three high-intensity local spots. It is observed that there are also transverse OAMs ${j}_{\mathrm{y}}$ or ${j}_{\mathrm{z}}$ with the same value. This distribution is consistent with the LG light^50^. Thus, although the combined field is not an ideal vortex light, it has a radial OAM distribution similar to that of LG light. Furthermore, we calculate the OAM of the local spot A with respect to the mass axis of the local spot A, ${j}_{\mathrm{x}}$ is close to 0; that is, there is no longitudinal OAM for a single local spot, which means that there is no “twist.” This characteristic is also consistent with that of the LG light. In summary, for a single local spot, there is no “twist” (${j}_{\mathrm{x}}$ ≈ 0); however, the four local spots as a whole show that there is a spiral structure relative to the center of the entire beam (${j}_{\mathrm{x}}$ = 1).

Moreover, from the intensity isosurface distribution of the high-intensity local spots shown in Fig. 3b, it can be clearly observed that the spots spatially propagate stably along the *x*-axis within the Rayleigh distance.

**Figure 4:**
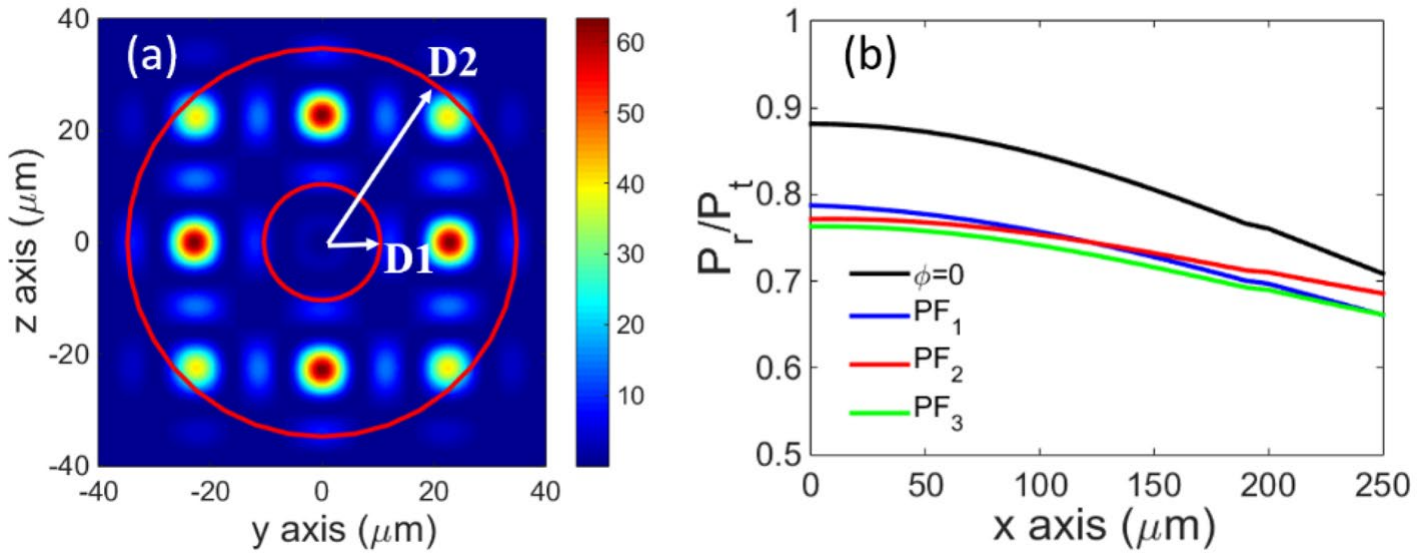
(**a**) Schematic of the ring used for the power-ratio calculation, where *D*1 and *D*2 represent the inner and outer ring radii, respectively. (**b**) The evolution of the ratio of the power in the ring ${p}_{\mathrm{r}}$ to the total combined field power ${p}_{\mathrm{t}}$ with the propagation distance in the cases of different random phases. ${PF}_{1}$(phase fluctuation), ${PF}_{2}$, ${PF}_{3}$ represent three random phase fluctuation processes.

Furthermore, the power distribution of the combined field was also checked according to different relative phases of the sub-beams. Owing to the intensity distribution characteristics of the vortex beam, we only consider the power in the ring between *D*1 and *D*2; *D*1 and *D*2 are the inner and outer radii of the ring, respectively, as shown in Fig. 4a. The values of *D*1 and *D*2 are taken as the inner and outer radii of the sub-beam, where the intensity is half of the peak intensity. The following formula is adopted:

9 $$\begin{array}{*{20}c} {\eta = \frac{{\mathop \smallint \nolimits_{D1}^{D2} \mathop \smallint \nolimits_{0}^{2\pi } I\left( {r,\varphi ,x} \right)}}{{\mathop \smallint \nolimits_{0}^{\infty } \mathop \smallint \nolimits_{0}^{2\pi } I\left( {r,\varphi ,x} \right)}},} \\ \end{array}$$

where $\eta$ is the combined field synthesis efficiency, which is defined as the power ratio of power ${p}_{\mathrm{r}}$ in the ring to the total combined field power ${p}_{\mathrm{t}}$ at the cross section. $I(r,\varphi ,x)$ is the intensity distribution of the cross-section of the combined beam. The results of the power ratio for different random phases are shown in Fig. 4b. The black line represents the power ratio when the sub-beams carry zero random phase, that is, $\phi =0$. The largest power ratio (88%) appears at the waist plane at *x* = 0. With an increase in the distance between the observation and waist planes, the power ratio decreases slightly but remains above 70%. Considering that phase fluctuation may occur in the actual experiment, we also calculate the power ratio after adding a random phase that varies in the interval [0, 2$\pi$] to each sub-beam; that is, in Eq. (1), $\phi$ is not equal to zero for each sub-beam at the waist plane. The numerical calculation that considers three times the phase fluctuation shows that the trends of the four curves are almost the same, and there is no clear difference between different phase fluctuations, as demonstrated in Fig. 4b. However, the power ratio after adding the random phase decreases slightly compared with the case of *ϕ* = 0, especially at the waist plane, which implies that the distribution of energy tends to be dispersed after adding the random phase.

**Figure 5:**
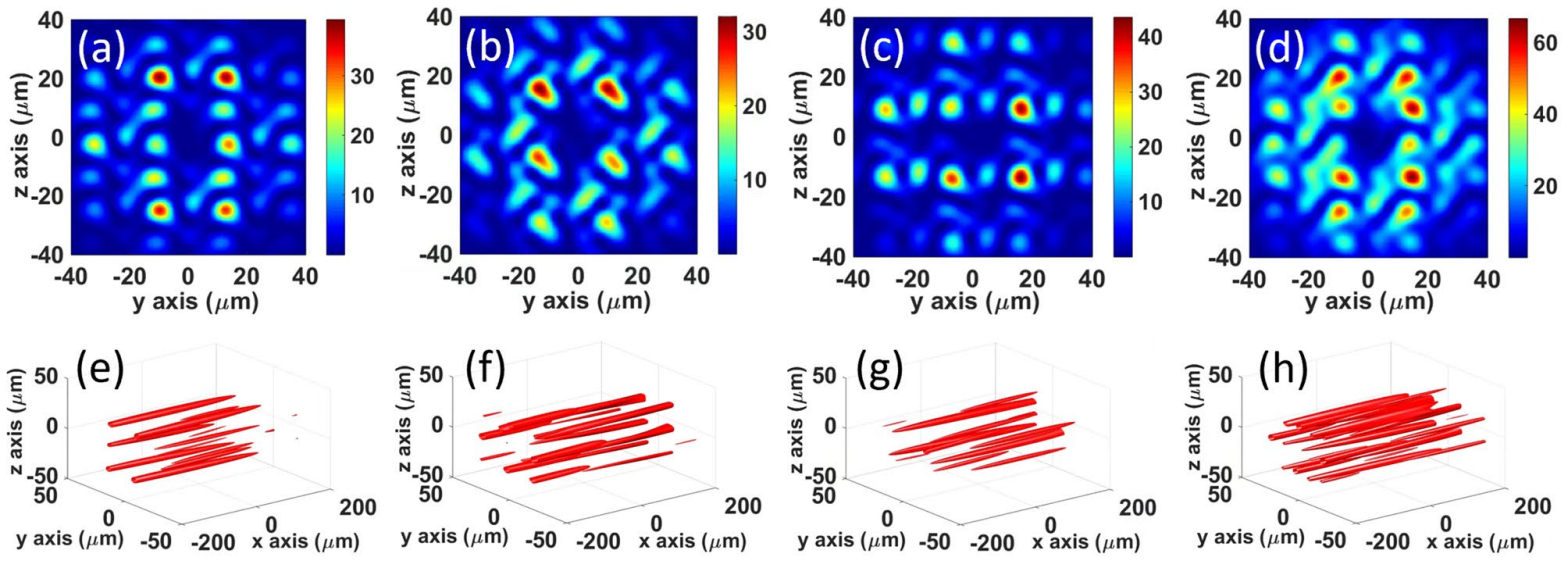
(**a**)–(**c**) Intensity distribution of the combined field at the waist plane with different random phases. (**e**)–(**g**) Isosurface distribution corresponding to (**a**)–(**c**) within the Rayleigh distance filtered with the isosurface value ${I}_{\mathrm{max}}\cdot 0.5$. ${I}_{\mathrm{max}}$, which is the peak intensity of the combined field in (**a**)–(**c**). (**d**), (**h**) Additive intensity distribution and isosurface of the above three random results. See Supplementary Fig. S2a–d for the phase diagram of (**a**–**d**), respectively.

Moreover, we note that the intensity distribution of the combined field at the waist plane and the propagation of the high-intensity spots near the waist plane change significantly after adding a random phase to the sub-beams, as shown in Fig. 5. The top row of the figure shows the intensity distribution of the combined field. We observe that the peak intensity decreases and more high-intensity spots appear. The bottom row shows the isosurface of the high-intensity spots within the Rayleigh distance. It can be observed that, owing to the random phase, the high-intensity local spots stably propagate over a shorter distance. The random phase clearly affects the distribution of the combined field; however, the intensity of the spots is still higher than that of the surrounding domain, which indicates that the interference effect is still strong. It is known that the existence of a random phase suppresses stimulated Raman scattering and filamentation when a laser beam propagates in plasma. In the present case, the existence of a random phase will further broaden the OAM spectrum of the combined field, which is beneficial for the suppression of the intense vortex laser–plasma interactions.

**Figure 6:**
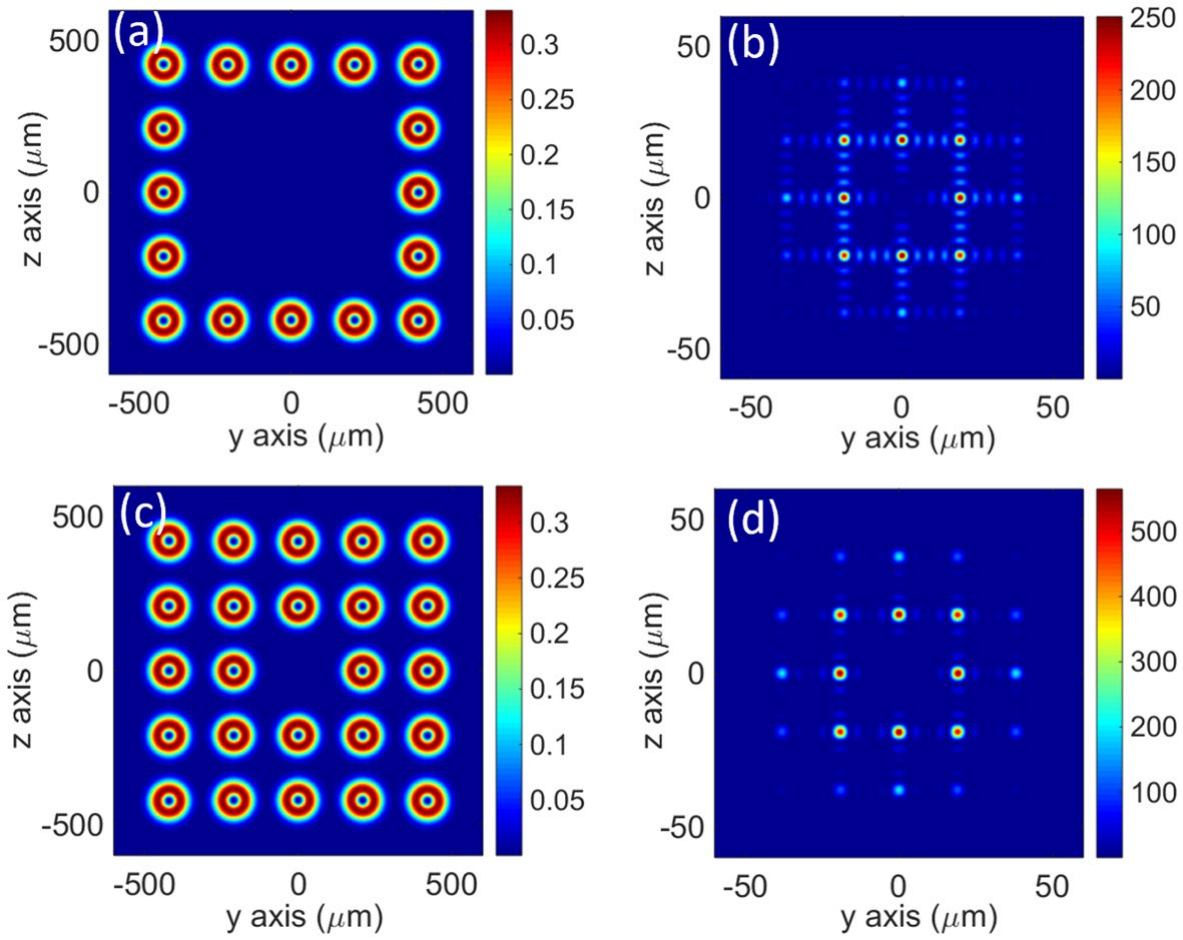
Numerical results when the number of the incident sub beams *N* = 16, 24. The peak intensity ${I}_{0}$ of the sub-beams is set to 1 at the waist plane *x* = 0. (**a**), (**c**) Intensity distributions of the sub-beams at the source plane *x* = − 4 mm when *N* = 16, 24, respectively. (**b**), (**d**) Intensity distributions of vortex light combined field at the waist plane *x* = 0, when *N* = 16, 24, respectively.

It should be noted that our proposed synthesis method is also suitable for the synthesis of more beams. For example, the intensity distribution at the waist plane of the combined field has been analyzed for the cases of the 16 and 24 sub-beams (*N* = 16, 24), as shown in Fig. 6. It can be observed that when the number of incident sub-beams increases, there are still high intensity local points at the waist plane of the combined field. Yet, the number of high-intensity local points symmetrically distributed around the central point becomes more. The peak intensity of the combined field for the cases of 16 and 24 sub-beams is approximately 250${I}_{0}$ and 564${I}_{0}$, respectively, which is almost the highest intensity of each high-intensity regions. Similarly, the peak intensity of the combined field is also close to the perfectly coherent intensity (${N}^{2}{I}_{0}$) in the case of multiple beams. However, the synthesis of more beams means a larger area in the source field, which will be difficult for practical experiments. At the same time, phase control of sub-beams will be more difficult.

In addition, we know that a continuous vortex can be described as $E={r}^{\left|l\right|}\mathrm{exp}(il\varphi )$, where *E* is the field, and *r* and $\varphi$ are the polar coordinates^51^. The intensity is continuous in a donut-like around the center with a non-zero phase circulation in multiplication of 2π. Compared with continuous vortices that have continuous phase function over the contour, a discrete vortex consists of a finite number of sites in a cyclic array, where the intensity in the center is zero, and phase circulates from one site to the next in either a vortex or anti-vortex direction^52^. Unlike a continuous vortex, the discrete vortex consists of step-like behavior of phase along the discrete contour encompassing the phase singularity^53^. For our case, the incident sub beam is LG light, which belongs to continuous vortex, but the distribution of the combined field is similar to that of the discrete vortex field.

## Conclusion

In this study, the coherent superposition of multiple LG beams with oblique incidence was considered. The combined field generated in this scheme has ultra-high-intensity local spots, and the peak intensity is close to ${N}^{2}{I}_{0}$, when all the sub-beams are perfectly coherent. This is expected to produce relativistic vortex laser in practical experiments. Notably, most of the energy in the combined field is concentrated in high-intensity spots that remain spatially stable in the Rayleigh distance. The spatial distribution of the OAM in the combined field is similar to that of the incident LG light. This vortex field with both OAM and strong spot distribution provides a new development opportunity for intense vortex laser–plasma interaction. In addition, we consider the influence of the random phase between the incident sub-beams on the combined field, owing to the inevitable phase fluctuation between the sub-beams in the actual experiment. It was observed that the intensity of the combined field presents a slightly discrete distribution, which is promising to play an important role in the field of large-scale nuclear fusion, such as suppressing stimulated Raman scattering and filamentation when a laser beam propagates in plasma.

## Supplementary Information

Supplementary Information 1.

Supplementary Video 1.

Supplementary Information 2.

## Data Availability

All data generated or analyzed during this study are included in this published article_ENREF_51.
