# Supplementary material for: Intense vortical-field generation using coherent superposition of multiple vortex beams
Source: Sci Rep. 2023 Jan 20;13:1104. doi: 10.1038/s41598-023-28216-9 (PMC9859784; doi:10.1038/s41598-023-28216-9)
Supplement: Supplementary file 1 — Supplementary Information 1. [file 41598_2023_28216_MOESM1_ESM.docx]

Intense vortical-field generation using coherent superposition of multiple vortex beams: supplementary Material

Xinju Guo^1^, Xiaomei Zhang^1, #^, Dirui Xu^1^, Weixin Chen^1^, Yi Guo^1^, Ke Lan^2,3^, and Baifei Shen^1, *^

^1^*Department of Physics, Shanghai Normal University, Shanghai 200234, China*

^2^*Institute of Applied Physics and Computational Mathematics, Beijing 100094, China*

^3^*HEDPS, Center for Applied Physics and Technology, and College of Engineering, Peking University, Beijing 100871, China*

[^#^zhxm@shnu.edu.cn](mailto:#zhxm@shnu.edu.cn)

[^*^bfshen@shnu.edu.cn](mailto:*bfshen@shnu.edu.cn)

This supplement is composed of two sections, A and B. Section A shows the phase diagram corresponding to Fig.2 and Fig.5 in the manuscript. Section B shows the calculation process of orbital angular momentum results (OAM) in Table 1 in the manuscript.

**A. The phase diagram**


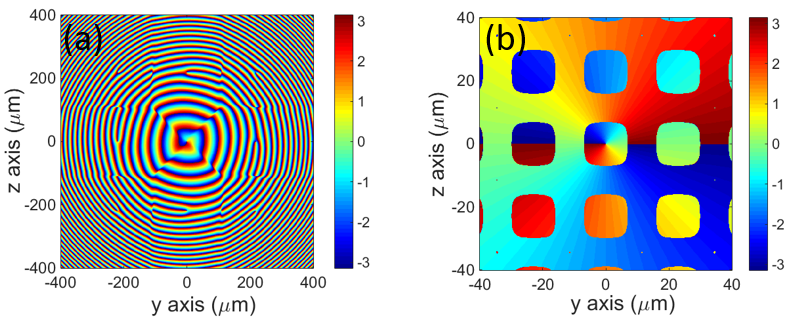


Fig. S1. Phase diagram corresponding to Fig.2. The unit of color bar is rad. (a) Phase distributions of the sub-beams at the source plane *x* = -5 mm. (b) Phase distributions of vortex light combined field at the waist plane *x* = 0.


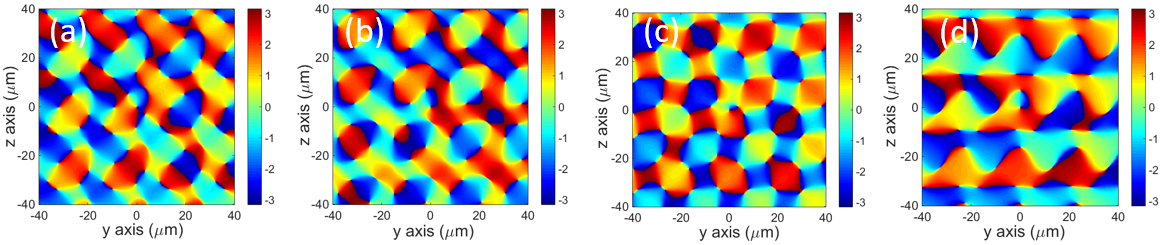


Fig. S2. Phase diagram corresponding to Fig.5. (a)–(c) Phase distribution of the combined field at the waist plane with different random phases. (d) Additive Phase distribution and isosurface of the above three random results. (a)(b)(c)(d) correspond to (a)(b)(c)(d) in Fig. 5, respectively.

**B. The calculation process of the orbital angular momentum**

In the OAM calculation part, we give the corresponding calculation process: in the analytical calculation, we give the vector equation of linearly polarized electromagnetic field:

$$\begin{aligned} \boldsymbol{A}=u\left( x,y,z \right)\cdot\exp\left( ikx \right)\boldsymbol{\#}\left( S1 \right) \end{aligned}$$

From the vector potential, the complex amplitude distribution of electric and magnetic field components can be obtained:

$$\begin{aligned} \boldsymbol{B}=ik\left( u\hat{\boldsymbol{z}}+\frac{i}{k}\frac{\partial u}{\partial z}\hat{\boldsymbol{x}} \right)\cdot e^{ikx}\boldsymbol{\#}\left( S2 \right) \end{aligned}$$

$$\begin{aligned} \boldsymbol{E}=i\omega\left( u\hat{\boldsymbol{y}}+\frac{i}{k}\frac{\partial u}{\partial y}\hat{\boldsymbol{x}} \right)\cdot e^{ikx}\boldsymbol{\#}\left( S3 \right) \end{aligned}$$

Considering the paraxial limit, the derivative of the field in the *z* direction is ignored.

Based on the analytically calculated values of electric field ***E*** and magnetic field ***B***, we use $\boldsymbol{J}=\int_{V} (\boldsymbol{r}\times\boldsymbol{p}) dV$ to calculate the OAM of the combined field, where ***J*** is the angular momentum of the electromagnetic field, *r* is the polar coordinate position of the *y-z* plane, and ***p*** is the momentum density of the electromagnetic field, $\boldsymbol{p}=\varepsilon_{0}\boldsymbol{E}\times\boldsymbol{B}$, $\varepsilon_{0}$ is the vacuum dielectric permittivity, and *V* is the whole electric field calculation region.

The OAM in different directions of the synthetic field is:

$$\begin{aligned} J_{x}=\int_{V} \left( \boldsymbol{x}\times\boldsymbol{p} \right)dV\#\left( S4 \right) \end{aligned}$$

$$J_{y}=\int_{V} (\boldsymbol{y}\times\boldsymbol{p}) dV$$

$$J_{z}=\int_{V} (\boldsymbol{z}\times\boldsymbol{p}) dV$$

At the same time, in order to compare with the OAM of the incident sub beam, we average the OAM to each photon and normalize it to $\hbar$.

$$\begin{aligned} j_{x}=(J_{x}/n)/\hbar\#\left( S5 \right) \end{aligned}$$

$$j_{y}=(J_{y}/n)/\hbar$$

$$j_{z}=(J_{z}/n)/\hbar$$

where *n* is the number of photons in the combined field.
