# Supplementary material for: Intense vortical-field generation using coherent superposition of multiple vortex beams
Source: Sci Rep. 2023 Jan 20;13:1104. doi: 10.1038/s41598-023-28216-9 (PMC9859784; doi:10.1038/s41598-023-28216-9)
Supplement: Supplementary file 3 — Supplementary Information 2. [file 41598_2023_28216_MOESM3_ESM.docx]

Intense vortical-field generation using coherent superposition of multiple vortex beams

Xinju Guo^1^, Xiaomei Zhang^1, #^, Dirui Xu^1^, Weixin Chen^1^, Yi Guo^1^, Ke Lan^2,3^, and Baifei Shen^1, *^

^1^*Department of Physics, Shanghai Normal University, Shanghai 200234, China*

^2^*Institute of Applied Physics and Computational Mathematics, Beijing 100094, China*

^3^*HEDPS, Center for Applied Physics and Technology, and College of Engineering, Peking University, Beijing 100871, China*

[^#^zhxm@shnu.edu.cn](mailto:#zhxm@shnu.edu.cn)

[^*^bfshen@shnu.edu.cn](mailto:*bfshen@shnu.edu.cn)

The video of Supplementary Material 2: The intensity distribution of combined field at different cross sections. These the four local spots appear and gradually strengthen within the Rayleigh distance of the combined field. The energy keeps to accumulate towards four local spots during the propagation, which can be observed from the evolution process of the combined field.

The supplementary video should play on any mainstream video player.
